# Succinic Semialdehyde Dehydrogenase Deficiency: An Update

**DOI:** 10.3390/cells9020477

**Published:** 2020-02-19

**Authors:** Miroslava Didiasova, Antje Banning, Heiko Brennenstuhl, Sabine Jung-Klawitter, Claudio Cinquemani, Thomas Opladen, Ritva Tikkanen

**Affiliations:** 1 Institute of Biochemistry, Medical Faculty, University of Giessen, Friedrichstrasse 24, 35392 Giessen, Germany; Miroslava.Didiasova@biochemie.med.uni-giessen.de (M.D.); Antje.Banning@biochemie.med.uni-giessen.de (A.B.); 2 Division of Neuropediatrics and Metabolic Medicine, Department of General Pediatrics, University Children’s Hospital Heidelberg, 69120 Heidelberg, Germany; Heiko.Brennenstuhl@med.uni-heidelberg.de (H.B.); Sabine.Jung-Klawitter@med.uni-heidelberg.de (S.J.-K.); Thomas.opladen@med.uni-heidelberg.de (T.O.); 3 SSADH-Defizit e.V., Leipziger Platz 5, 50733 Cologne, Germany; info@ssadh.de

**Keywords:** succinic semialdehyde dehydrogenase deficiency, gamma-amino butyric acid, organic acidurias, enzyme replacement therapy, pharmacological chaperones, clinical trials, autophagy

## Abstract

Succinic semialdehyde dehydrogenase deficiency (SSADH-D) is a genetic disorder that results from the aberrant metabolism of the neurotransmitter γ-amino butyric acid (GABA). The disease is caused by impaired activity of the mitochondrial enzyme succinic semialdehyde dehydrogenase. SSADH-D manifests as varying degrees of mental retardation, autism, ataxia, and epileptic seizures, but the clinical picture is highly heterogeneous. So far, there is no approved curative therapy for this disease. In this review, we briefly summarize the molecular genetics of SSADH-D, the past and ongoing clinical trials, and the emerging features of the molecular pathogenesis, including redox imbalance and mitochondrial dysfunction. The main aim of this review is to discuss the potential of further therapy approaches that have so far not been tested in SSADH-D, such as pharmacological chaperones, read-through drugs, and gene therapy. Special attention will also be paid to elucidating the role of patient advocacy organizations in facilitating research and in the communication between researchers and patients.

## 1. Succinic Semialdehyde Dehydrogenase Deficiency: Clinical Phenotype, Genetics, and Standard Care

### 1.1. Clinical Phenotype and Diagnosis of Succinic Semialdehyde Dehydrogenase Deficiency

Succinic semialdehyde dehydrogenase deficiency (SSADH-D, also called 4-hydroxybutyric aciduria, OMIM #271980) is an ultra-rare monogenic disorder of the γ-amino butyric acid (GABA) metabolism, with approximately 450 patients known to the literature (reviewed in [1,2]). The first case of SSADH-D was identified in 1981 in a patient excreting γ–hydroxybutyric acid (GHB) in the urine, and only two years later, the deficiency of succinic semialdehyde dehydrogenase (SSADH) activity was demonstrated to be the underlying cause [3,4]. Variants of the *ALDH5A1* gene have later been shown to be the cause of SSADH-D [5], which is inherited in an autosomal recessive fashion. Heterozygous carriers of one defective allele show no clinical signs of the disease, whereas the patients who are either homozygous or compound heterozygous for disease-causing variants are affected to a varying degree. Enzymatic dysfunction of SSADH leads to an accumulation of potentially neurotoxic metabolites, including GABA and GHB, as well as numerous other substances (see Table 1). Despite ambitious scientific effort, detailed knowledge about many aspects of the pathophysiology of the underlying enzyme defect is still lacking, and at present, no curative treatment that would directly target the enzyme deficiency is available for SSADH-D. As with many other rare disorders affecting the central nervous system (CNS), several symptomatic treatments have been and are currently investigated [6,7].

The clinical picture of SSADH-D is highly heterogeneous, and in many cases, the somewhat nonspecific nature of the symptoms may delay the diagnosis of patients without prior family history of the disease [1,9]. However, common manifestations of SSADH-D include a varying degree of mental retardation, psychiatric disorders, autism-like symptoms, and impaired speech, along with sleep disturbances [1,10,11,12]. Some degree of developmental delay and intellectual disability are found in all patients, while around 80% of the patients are affected by ataxia and muscular hypotonia [9]. Starting in late childhood, most patients (around 60%) develop epileptic seizures, ranging from absence seizures to generalized forms of epilepsy, which are also present in adult patients.

Apart from the seizure phenotype, the disease usually does not exhibit a further progressive course. However, the highly variable clinical presentation of SSADH-D and the very poor genotype/phenotype correlation make diagnosis difficult [13]. Even in a single family with two or more affected children harboring the same pathogenic variants of SSADH, the degree of disability and the symptoms can vary greatly [14]. The exact cause of this variability despite the same genetic background is not known. Currently, there is active research to better understand the causative relationship between the molecular defect and the subsequent clinical consequences. In addition, the detailed molecular consequences of specific SSADH disease-causing variants in terms of SSADH enzyme function are a subject of active research.

**Figure 1:**
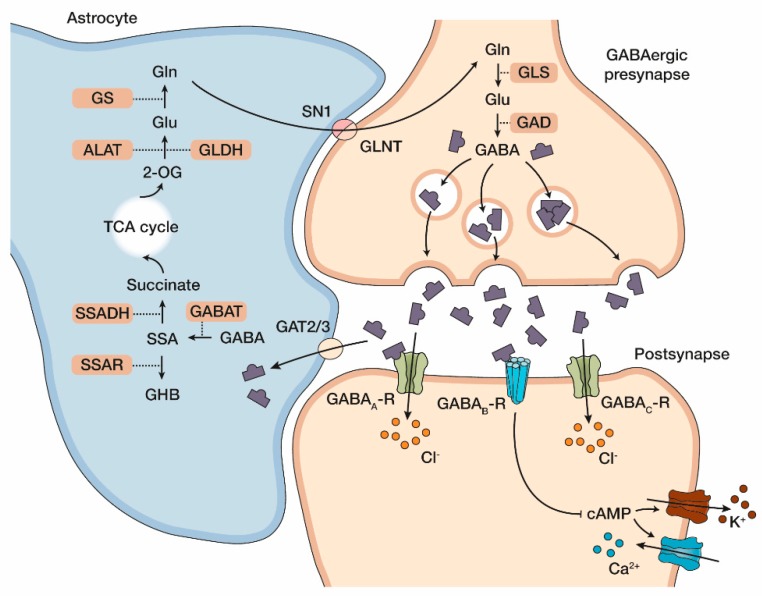
Overview of the synaptic cleft and the metabolic synopsis of a GABAergic synapse. The glutamate/GABA-glutamine cycle is depicted. GABA is synthesized in the presynaptic GABAergic synapse from glutamate (Glu) by glutamate decarboxylase (GAD) and is then packaged into vesicles. Upon electrophysiological activation, GABA is released into the synaptic cleft where it can bind to three known receptors. GABA_A_ and GABA_C_ receptors represent ionotropic receptors, whereas the GABA_B_ receptor is G-protein coupled and functions via adenylate cyclase or by direct coupling with other ion channels. GABA neurotransmission is terminated after uptake of GABA by GABA transporter 2/3 (GAT 2/3) into astrocytes, where GABA transaminase (GABAT) converts it into succinic semialdehyde (SSA). SSA is then oxidized by SSADH to succinate and serves as a substrate within the tricarboxylic acid (TCA) cycle. α-ketoglutarate (2-OG) can be used for the synthesis of Glu by alanine transaminase (ALAT) and glutamate dehydrogenase (GLDH) and glutamine (Gln) by glutaminase (GS). Gln is then shuttled back to presynaptic GABAergic neurons via glutamine transporter SLC38A3 (SN1) and glutamine transporter SLC38A2 (GLNT). In SSADH-D, SSA cannot be converted to succinate but is reduced to GHB by SSA reductase (SSAR) (adapted from [15,16]).

SSADH-D is caused by a defect in the catabolism of GABA, the main inhibitory neurotransmitter of the CNS. Figure 1 shows an overview of a GABAergic synapse and GABA metabolism. Excess GABA is usually removed by the successive action of several enzymes that mediate its degradation. GABA transaminase first removes the amino group of GABA, producing succinic semialdehyde (SSA). This metabolic product is turned over by the SSADH enzyme that converts it into succinic acid, which can be further metabolized in the tricarboxylic acid cycle. In SSADH-deficient patients, the GABA metabolic pathway is disrupted due to low or absent activity of SSADH. Due to this, SSA cannot be eliminated through its normal catabolic pathway and it is converted to GHB by an aldo-keto-reductase, resulting in elevated GHB levels.

In SSADH-D, a high degree of accumulation of GABA, GHB, and further metabolites is observed in the CNS as well as in the peripheral tissues and body fluids of the patients [13]. Table 1 shows a summary of some of these metabolites in the urine, plasma, and cerebrospinal fluid of SSADH-D patients. However, it is not clear which ones of these neuroactive substances are the main contributors to the neuronal impairment observed in SSADH-D, since a large excess of many of these compounds can exert neurotoxic effects. In fact, GHB is also known as a “rape drug”, as it can be misused to induce amnesia, which has led to considerable forensic interest towards GHB. In the nervous system, GHB can act by binding to its own receptor, but it has been shown that GHB also binds to certain GABA_A_ receptors [17], whereas GABA itself acts on GABA_A_, GABA_B_, and GABA_C_ receptors [18]. Thus, GABA receptors have been a target of research aiming at clinical interventions in SSADH-D by applying receptor agonists or antagonists. In addition to its role in the CNS, GABA has also been suggested to play a role in non-neuronal tissues by modulating endocrine functions and T lymphocyte proliferation and differentiation [19,20]. Thus, enzymes, such as SSADH, that control GABA levels may have an important role in the regulation of functions beyond neurotransmission. It is out of scope of this review to discuss the function of GABA as a neurotransmitter, and we would like to recommend the reviews by Petroff and Siucinska to interested readers [21,22]. However, clinical and pre-clinical interventions of GABA and GHB receptors in SSADH-D will briefly be discussed in the present review.

The diagnosis of SSADH-D is presently based on the detection of GHB in the organic acid profile in urine [13]. In addition, GHB can be measured in other body fluids [23,24]. Genetic testing should always be used to confirm biochemical findings that point to SSADH-D. SSADH-D can present as an autism spectrum disorder [25], and the *ALDH5A1* gene has been included in the commercially available autism gene panels [26]. Applying such panels to autism cases of an unknown etiology could thus help to identify new cases of SSADH-D, since many SSADH-D cases are likely to remain undiagnosed, as also suggested by the inhomogeneous global distribution of diagnosed cases [27]. The diagnostic rate could also be improved, e.g., by introducing newborn screening programs for organic acidurias, since it has recently been shown that the diagnosis of SSADH-D may be possible from a dried bloodspot [28].

### 1.2. Standard Care and Emerging Picture of the Pathophysiology in SSADH-D

At present, there is no curative treatment for SSADH-D that directly addresses the enzyme defect, although some treatment options are starting to emerge (see Section 3.1). Therefore, the patients mainly receive treatments aiming at ameliorating some of the symptoms. This is highly unsatisfactory for both patients and clinicians, and novel therapeutic approaches for treating the actual cause of the disease are needed. Conventional treatment approaches include speech, occupational, and physiotherapy, whereas with older patients, psychotherapy is also frequently applied. In patients suffering from epileptic seizures, one major treatment goal is seizure control [10]. A broad spectrum of antiepileptic medications has been used in patients with SSADH-D, including the sodium channel blockers lamotrigine and carbamazepine [6]. Magnesium valproate has shown efficacy in seizure control in one adolescent patient, but it has also been described to inhibit residual SSADH activity [29,30]. For vigabatrin, which is an irreversible inhibitor of GABA transaminase and thus inhibits the subsequent formation of SSA, inconsistent results and severe side effects have been shown (see Section 3.2 and [31,32]). In addition to these drugs, antidepressants and substances against anxiety and inattention are also used [11,33]. However, most of these agents are designed to act only against specific symptoms and do not address the actual pathomechanisms in SSADH-D.

Due to the role of SSADH in GABA metabolism, it was originally suggested that the clinical phenotype is mainly based on a fundamental imbalance in the amount of GABA, the major inhibitory transmitter of the CNS, and the toxic GHB. In fact, both substances can be found elevated in white and grey brain matter as well as in the urine and plasma of SSADH-D patients [34]. Recently, it has also become evident that further molecular events may damage specific cellular functions and thus contribute to the clinical picture. For example, in vitro experiments and the murine model have pointed to a redox imbalance, mitochondrial dysfunction, and altered signaling through the mTOR (mechanistic target of rapamycin) pathway in SSADH-D. In addition, alterations in the expression of numerous genes have been shown, which might contribute to the phenotype, e.g., by affecting ion efflux/influx in neuronal cells. In Chapter 3, we will summarize these findings and elucidate their potential therapeutic relevance in SSADH-D.

## 2. The *ALDH5A1* Gene and the SSADH Enzyme: Lessons Learned from Disease Models

### 2.1. ALDH5A1 Gene Splicing Isoforms and Genetic Variants in SSADH-D

**Figure 2:**
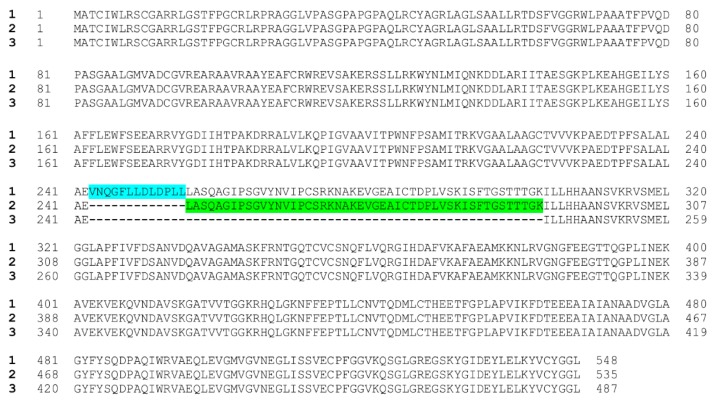
SSADH isoform protein sequences. Numbers to the left in bold show the isoform numbers. Blue highlight marks the 13 extra amino acids in isoform 1, whereas green highlight depicts the 48 amino acids present in isoforms 1 and 2 but missing from isoform 3.

The *ALDH5A1* gene resides in the human chromosomal region 6p22 [5,35], and the gene structure was characterized almost 20 years ago [36]. According to databases, such as GenBank, three human isoforms that show differences in the coding region have been predicted to exist. These isoforms arise due to alternative splicing of two in-frame exons in the pre-mRNA, and the isoforms 1 (NM_170740.1) and 2 (NM_001080.3) differ from each other in length by 39 bases (13 amino acids), with isoform 1 being the longer one. Isoform 3 (NM_001368954.1) has been predicted to lack a further 144 bases (48 amino acids) within the coding region, and it thus represents the shortest of the three isoforms. Figure 2 shows the predicted protein sequences of the three isoforms. According to our experience, isoform 2 mRNA appears to be the major form that is present in most cells, whereas we have found only trace amounts of isoform 1 mRNA, as detected by quantitative real-time PCR (M.D., A.B., and R.T., unpublished findings). To our best knowledge, no experimental evidence has been put forward that supports the existence of the predicted splicing isoform 3, neither at the mRNA nor at the protein level. The additional 13 amino acids in the isoform 1 translation product have been postulated to result in an inactive SSADH enzyme [36], and the same is likely to be the case for isoform 3, in which 48 amino acids would be missing from the cofactor binding domain of SSADH [37]. Thus, isoform 2 should be considered as the major enzymatically active form of SSADH in humans.

Various types of gene variants, including missense, nonsense and splicing mutations, deletions, and insertions, in the *ALDH5A1* gene have been shown to be responsible for SSADH-D (see Section 2.3 and [38,39]). By 2016, as many as 45 pathogenic variants were described, and further mutations are identified as more patients receive a correct diagnosis [31]. In many families (about 40% of the cases), homozygous gene defects are found due to consanguinity of the parents [40]. In addition to known pathogenic variants, various single nucleotide polymorphisms (SNPs) of unknown significance have been described in the respective databases (see, e.g., [41]).

Unfortunately, major confusion can be caused upon diagnosis by the use of different gene isoforms for the numbering of the detected variants. Many diagnostic companies base their numbering on the isoform 1, which is not the major splicing isoform of SSADH. Therefore, the numbering of all amino acids beyond Glu242 (the site of insertion of the additional 13 amino acids) is shifted by 13, and care has to be taken when analyzing the consequences at the amino acid level. In this review, the numbering of the genetic and protein variants is based on the isoform 2 and the protein translated from it.

### 2.2. SSADH Enzyme Function and Structure

**Figure 3:**
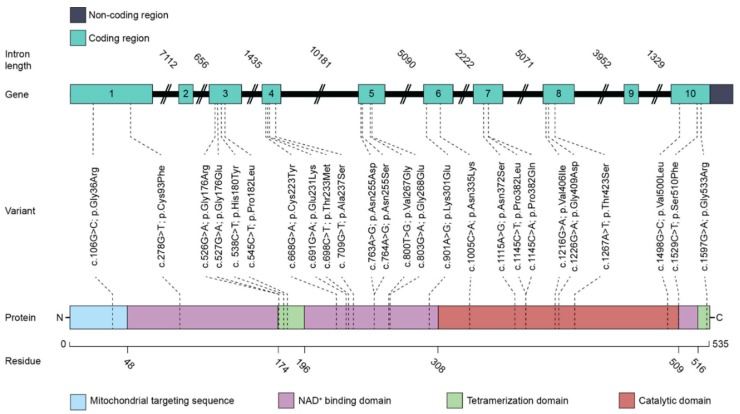
Exon and domain structure of SSADH, and localization of selected SSADH-D missense variants. The human SSADH isoform 2 exhibits 535 amino acids. Green boxes show the exons, joined by the black lines representing introns, the length of which is given above the lines. Colored blocks refer to the protein domains as indicated. Numbers below the bar refer to the first amino acid of the respective domain. Dashed lines connect the selected disease-causing variants with the respective exons and protein domains.

The SSADH enzyme precursor polypeptide of 535 amino acids (isoform 2) is synthesized by the cytosolic free ribosomes. This polypeptide contains a predicted mitochondrial targeting sequence (MTS) of 47 amino acids in its amino terminus (Figure 3). This MTS has been suggested to be responsible for the mitochondrial matrix targeting of the SSADH precursor, although experimental evidence has so far not been presented. In the mitochondrial matrix, SSADH folds and forms oligomers that represent the enzymatically active form [42]. It is generally accepted that SSADH is a tetrameric protein, even though a trimeric structure has been suggested but was later shown to be incorrect [43,44].

The three-dimensional structure of the recombinant human SSADH enzyme (amino acids 48-535) has been solved with a resolution of 2.0 Å [42]. Each asymmetric unit of the crystal structure contained the monomeric form of the polypeptide, but a tetrameric structure was predicted for SSADH by applying the crystallographic F432 symmetry model. Three domains that are responsible for the cofactor NAD^+^ binding (amino acids 48–173, 196–307, and 509–524), for the catalysis (amino acids 308-508), and for the tetramerization (amino acids 174–195 and 525–535) can be identified in each monomeric structure ( [42], see also Figure 3). The first two domains mainly exhibit α-helical and β-sheet structures, whereas the tetramerization domain contains three β-strands in antiparallel orientation.

SSADH is a member of the aldehyde dehydrogenase (ALDH) protein family, and its 3D structure shows a clear homology to other known members of this family. However, the active site and the substrate entrance exhibit features that are specific to SSADH. This is consistent with the fact that, as compared to other members of the ALDH family, SSADH shows a remarkable substrate specificity towards SSA. Kim and coworkers showed that the accessibility of the SSADH active site appears to be mediated by a redox-switch mechanism, which may explain the high substrate specificity of SSADH [42]. In the crystal structure of SSADH that was obtained in the absence of a reducing agent, the catalytic Cys340 within the active site appears to be engaged in a disulfide bond with Cys342. This results in a conformation that would prevent the entry of both the substrate SSA and the cofactor NAD^+^ to their respective binding sites, resulting in a catalytically inactive state of the enzyme. Exchange of the Cys342 residue prevented the formation of this closed conformation and resulted in a higher enzyme activity. The authors suggested that the activity of human SSADH might physiologically be controlled by oxidative stress and the redox state within the mitochondrial matrix. This is especially intriguing since changes in the redox status have been shown to be a feature of SSADH-D (reviewed in [37]), and these alterations might also contribute to the profound lack of residual activity that has been observed in the case of most of the disease-causing variants.

### 2.3. Consequences of the Pathogenic Variants Found in SSADH-D

Residual SSADH enzyme activity of pathogenic *ALDH5A1* variants has been investigated in several studies based mainly on overexpression of the variants (see, e.g., [8,38,39,45,46,47]). However, so far, it has not been possible to conclusively establish how each individual variant affects the enzyme activity in patients. As the residual enzyme activities may vary largely even among siblings with the same genotype, it is likely that further factors play a major role in regulating to which degree the genetic variants impair the SSADH enzyme activity and function. Identifying these factors would be of major interest, as this might provide a novel means of ameliorating the consequences of the lack of SSADH activity and facilitate the generation of novel therapies for SSADH-D. With technological progress that facilitates a more detailed analysis, more attention should be paid to the genotype/phenotype correlation, and both experimental and in silico approaches, such as modeling the structure of the pathogenic variants, should be used to elucidate how a specific genetic defect affects the function of the SSADH enzyme.

Numerous pathogenic variants localized throughout the SSADH polypeptide have been identified in SSADH-D patients. Most of these variants affect the predicted cofactor binding domain, but this does not necessarily imply that this domain is more vulnerable than other regions of SSADH. It may rather reflect the fact that the NAD-binding domain occupies almost half of the enzyme, with 254 of the 535 amino acids being part of this domain (see Figure 3). The structural and functional consequences of some of the disease variants have been analyzed, but most of them appear to have a very profound effect on the SSADH enzyme activity, at least when analyzed in an overexpression system (see, e.g., [36,38]). Table 2 summarizes some of the predicted and experimentally addressed consequences of SSADH-D missense variants (see also Figure 3).

Based on the crystal structure of SSADH, Kim and colleagues suggested that many of the missense variants that contain substitutions in an amino acid buried deep within the polypeptide (e.g., Cys93Phe, Cys223Tyr, Thr233Met, and Pro382Leu) could result in a profound destabilization of the enzyme structure [42]. Many other published variants (e.g., Gly176Arg, Gly176Glu, Pro182Leu, and Gly533Arg) are localized within the tetramerization domain [36,38,48], and the replacement of the small Gly residues by bulky and charged residues, such as Arg or Glu, is very likely to impair SSADH oligomerization and thus also its stability. Notably, many of the SSADH-D disease-causing variants are substitutions of Gly residues. It has been shown that such Gly residues are especially well conserved in the ALDH family of proteins [50]. Therefore, substitution of Gly residues in SSADH may be particularly deleterious in terms of its structure. Consistent with these predictions, our unpublished findings in patient fibroblasts exhibiting some of these variants show that the amount of the SSADH polypeptide detected by Western blot is highly reduced. Some SSADH-D variants may also affect substrate or cofactor binding and thus result in the impairment of the catalytic activity. For example, Gly268 is involved in the binding of the cofactor NAD^+^. Thus, the SSADH-D variant with a substitution Gly268Glu is likely to exhibit impaired NAD^+^ binding and thus shows a strong negative effect on the catalytic activity of SSADH [42].

In addition to the known SSADH-D variants, various SNPs that result in an amino acid exchange have been detected in the coding region of SSADH. For most of these variants, the significance and relevance for the enzyme function and activity are unknown. One exception is the His180Tyr variant (SNP rs2760118) whose consequences have been analyzed in an overexpression system [36,38]. When overexpressed, the His180Tyr SSADH mutant has 83% of wildtype activity, and should thus have no profound pathogenic relevance. However, when His180Tyr is expressed in combination (as a double mutation in the same construct or in the same allele in the case of patients) with another mild SSADH variant, such as Pro182Leu, it appears to reduce the residual enzyme activity of its counterpart even further [38]. Interestingly, His180 and Pro182 are localized in the oligomerization domain, so they may cause only a slight impairment of SSADH tetramerization when present alone. However, they appear to produce a more profound effect when combined with another SSADH variant. Therefore, despite their mild individual effect on SSADH enzyme activity, His180Tyr and Pro182Leu substitutions cannot be considered as harmless or completely non-pathogenic, and their relevance needs to be considered in the context of other substitutions that are present in the same allele. Unfortunately, according to our experience, the SNPs resulting in amino acid exchanges that are listed as non-pathogenic in the databases are frequently ignored and not disclosed by companies providing genetic diagnosis for SSADH-D. However, for the researchers and clinicians, it would be essential to know the exact genotypic variants present in a patient, in order to be able to correctly dissect the potential molecular consequences of the mutations. Thus, all sequence variants of the mutated gene should always be disclosed in the diagnosis files, as it is likely that many patients will have additional substitutions, such as His180Tyr, which has been shown to be present in more than 30% of the analyzed chromosomes within the GnomAD exome database [51].

Most of the studies addressing the consequences of SSADH-D gene variants have measured the residual enzyme activity only, without paying attention to the expression level, mRNA amount, or localization of the enzyme. Furthermore, much of the data are derived from overexpression systems. Therefore, in the future, it will be important to perform such studies in more suitable systems, especially in patient-derived cells, such as fibroblasts, or neuronal cells derived from induced pluripotent stem cells (see below). Furthermore, when using overexpression studies, the effect of specific variants should always be characterized in cells that lack endogenous SSADH. This is important since SSADH is a tetramer, and oligomerization of the mutant enzyme with the endogenous wildtype SSADH might not reveal the true degree of functional impairment of the variant, as compared to the expression of the variant alone. We have recently generated CRISPR-based knockout HEK293T cells that do not express endogenous SSADH, which will be a valuable tool to analyze the molecular consequences of SSADH mutations in a context without endogenous background (M.D., A.B., and R.T., unpublished data).

### 2.4. Disease Models: Cellular Models, Organoids, and the SSADH Knockout Mouse

The simplest models for studying SSADH-D are cell culture-based systems, either ectopic overexpression of the enzyme in a suitable cell line, such as HEK293 cells, or patient cells, e.g., dermal fibroblasts, that can easily be obtained and cultured. The advantages of these systems are the ease of use and low price of culture reagents. However, overexpression may result in aberrant behavior of the protein due to aggregation or saturation of the machinery required for the mitochondrial translocation. Dermal fibroblasts from a patient express low levels of SSADH, but they exactly represent the genotype of the patient. On the other hand, their life span is very limited, and finding a suitable isogenic control may be difficult. Therefore, modern genome editing methods, such as the CRISPR/Cas technology, can be used to produce genomic SSADH variants that mimic the patient genotype in a suitable cell line.

Patient-derived fibroblasts or peripheral blood mononuclear cells (PBMCs) can be reprogrammed to induced pluripotent stem cells (iPSCs). Great advances have been made in this technology in recent years, and it is becoming ever more popular as a model system to study human diseases. The advantage of this model is that iPSCs can be differentiated into all cell types of the body, especially those that represent the tissue of interest, such as the liver or the brain. This can be done in a two-dimensional culture in a dish, and established protocols are available for various cell types. Although differentiated cells, such as neurons, representing the patient genotype are highly desirable, the generation, culture, and differentiation of iPSCs is not trivial and requires expensive cell culture media and supplements. Recently, diverse protocols for the differentiation of iPSCs in 3D culture systems to so-called organoids have been published. These include protocols for the generation of cerebral organoids [52] but also regionalized brain organoids like midbrain or forebrain organoids [53,54,55]. These organoids usually consist of several cell types organized in structures resembling the organs in question. This method is highly sophisticated, and the organoids recapitulate the situation in patients to a higher degree than 2D cultures. However, the method is expensive and time-consuming. Therefore, organoids will surely be very useful in the future for studying diseases and testing therapies, but they are unlikely to completely replace the 2D culture systems. Moreover, they are not easy to use for high content screening approaches due to their high batch-to-batch variations and the difficulties in finding a suitable readout for screening purposes.

The murine SSADH knockout (*Aldh5a1*^−/−^) model was developed almost two decades ago by Hogema and colleagues [56]. These mice are well accepted as an SSADH-D animal model, although the phenotype is far more severe than what is seen in most human SSADH-D patients. The characteristics of these mice have recently been very thoroughly summarized by Kim et al. [37]. We thus kindly refer the readers to this excellent review, and here we only elaborate on the major features of this mouse model. The SSADH knockout mice develop generalized seizures by 3–4 weeks of life, resulting in an early death. The metabolic features associated with these mice well recapitulate those of the human SSADH-D patients, e.g., elevated GABA and GHB levels in tissues and in the urine, and absence seizures during the second week of life, which tend to culminate into more generalized seizures in the third week of life. Many of the findings observed in SSADH-D patients are found in this mouse model in an exacerbated form. Therefore, the SSADH knockout model currently represents the best available animal model that mimics the main features associated with the human disease.

Disruption of myelin formation is a typical feature connected with neurological dysfunction [57]. Even though myelination defects do not appear to be prominent in SSADH-D, and there are very few reports that point to myelination defects in SSADH-D patients [58,59], the *Aldh5a*^−/−^ knockout mouse revealed substantial changes in myelination, attributed to the dysregulated GABA/GHB neurotransmission. It was shown that the expression of myelination-associated genes was significantly downregulated in the hippocampus and cortex of these mice. Furthermore, a 30% decrease in ethanolamine phosphatide, a major plasmalogen myelin component, was documented [60]. Further studies revealed a decreased ethanolamine glycerophospholipid mass in *Aldh5a*^−/−^ mice [61]. Therefore, it would be justified to further investigate myelination changes in *Aldh5a*^−/−^ mice and to extend these studies to humans as well.

For potential treatment trials in the animal model, the early lethality poses a major problem as any treatment would have to be started very early (latest around day 10), where the pups are still quite small in size and may be difficult to handle. On the other hand, the extended survival beyond the expected lifetime of 3–4 weeks can be used to monitor the treatment effect. However, treatment trials to monitor long-term benefits are not possible. Therefore, an animal model that more precisely mimics the mainly slow course of the disease in humans would be highly desirable and could be obtained by transgenic or CRISPR/Cas-mediated knock-in of a suitable disease-causing genetic variant.

### 2.5. Mitochondrial Dysfunction, Redox Imbalance, and Autophagy Defects in SSADH-D

The lack of SSADH enzyme activity results in alterations in the amount of GABA, GHB, and other metabolites (see Table 1) that are likely to be directly relevant for the pathogenesis and the symptoms observed. However, indirect effects that result in organelle dysfunction or impairment of signaling have been suggested to contribute to SSADH-D. For example, oxidative damage has been shown to be a prominent feature in both patients and in the murine SSADH-D model, and mitochondrial aberrations are frequently observed ( [62] and reviewed in [37]). Interestingly, GHB has been shown to inhibit lipid biosynthesis and to induce oxidative stress in rats [63,64]. Silva and colleagues suggested that altered lipid amounts were not caused by a direct inhibition of the enzymes involved in lipid biosynthesis by GHB, whereas mitochondria were likely to contribute to this effect [64]. These findings again point to the importance of indirect toxic effects in the disease pathogenesis of SSADH-D.

A subset of SSADH-D patients shows rather non-specific signal changes in their brain magnetic resonance imaging (MRI). Signs of metabolic toxicity can be revealed by MRI of patient brains [9,65], pointing to an important contribution of oxidative damage. This is consistent with the findings in other organic acidemias, such as methylmalonic acidemia [66,67], showing that redox imbalance and mitochondrial dysfunction are also a typical feature in these diseases. Beyond that, aberrations in lipid biosynthesis have also been shown in organic acidemias [68], which may lead to aberrant brain development and myelination already pre- and postnatally. In the developing brain, lipids, such as gangliosides, are of vital importance for proper brain maturation and neurite outgrowth [69,70]. In the SSADH knockout mouse model, downregulation of several genes associated with myelination and reduced levels of major myelin plasmalogen components have been observed [60]. Thus, impairment of lipid homeostasis and myelination may also be a common feature in SSADH-D and other organic acidemias.

Several studies have addressed the role of oxidative stress/damage in SSADH-D. Using the mouse model, Latini and colleagues showed that the total radical-trapping antioxidative potential (TRAP) and glutathione (GSH), which represent the non-enzymatic antioxidative defense mechanisms in tissues, were highly reduced in some tissues, especially in the liver and in the cerebral cortex [71]. Low GSH levels have also been observed in an SSADH-D patient [62]. Latini and coworkers also observed an increased lipid peroxidation in the cerebral cortex and the liver of the SSADH knockout mice, demonstrating that oxidative damage to lipids takes place in these tissues [71]. Interestingly, SSADH has been shown to be the major enzyme that is required for the elimination of 4-hydroxy-trans-nonenal (HNE), a lipid peroxidation product, in the brain [72]. HNE has also been shown to be involved in the induction of oxidative stress in other diseases, such as Alzheimer’s disease [73,74]. In SSADH-D, it is thus likely that in the absence of SSADH activity, HNE may not be disposed of in a proper way, and its increased levels may further contribute to oxidative stress.

Lakhani and coworkers have shown that GABA is involved in the regulation of specific forms of autophagy, mitophagy, and pexophagy, by increasing the activation of the mTOR complex in a yeast model [74]. Autophagy is an important clearance process during which altered cell organelles, such as mitochondria (mitophagy) and peroxisomes (pexophagy), are eliminated via lysosomal uptake and degradation (for a review, see [75,76]). Impaired mitophagy and pexophagy as such can result in increased production of reactive oxygen species (ROS) and oxidative stress, which are important pathogenic features in SSADH-D. In line with these findings, SSADH-deficient mice exhibit increased numbers of mitochondria that show morphological defects, which would be consistent with impaired mitophagy [74]. Defects in autophagy are also associated with diverse neurodegenerative disorders, including the neuronal ceroid lipofuscinoses, Alzheimer’s disease, and Parkinson’s disease, which also show mitochondrial dysfunction and increased oxidative stress [75,77].

The activation state of the mTOR complex 1 (mTORC1) is a key element in the regulation of autophagy. The active mTORC1 is associated with lysosomes where it inhibits two major inducers of autophagy, the Atg1 kinase and the Atg13 protein. Nutrient starvation is associated with dissociation of the inactive mTORC1 from the lysosomes, resulting in increased autophagy. Thus, activation of mTOR by GABA is likely to exacerbate the accumulation of aberrant mitochondria in SSADH-D by inhibiting mitophagy, suggesting that mTOR inhibition may exhibit therapeutic potential in SSADH-D. Vogel and coworkers analyzed the effect of various mTORC inhibitors in SSADH knockout mice [78,79]. They showed that the lifespan of the mice can be extended by dual inhibitors of mTORC1 and mTORC2, Torin 2, and XL-765 [79]. In addition, the mTOR-independent autophagy-inducing peptide tat-Beclin 1 [80] also prevented early lethality in these mice [79]. These findings suggest that inhibition of mTOR activity and/or induction of autophagy may exhibit therapeutic potential in SSADH-D.

Modulation of autophagy is expected to show high potential for diseases as diverse as metabolic disorders, cancers, neurodegenerative, and infectious diseases [81,82], and drugs that modulate the autophagic flux and exhibit therapeutic potential for various diseases have been identified in drug screens [82]. Disease-specific autophagy drug screens may also reveal novel pathways to be targeted for autophagy induction, as has recently been shown for juvenile neuronal ceroid lipofuscinosis, where pathways, such as calcium signaling, isoprenoid pathway, and microtubule dynamics, were identified as potential drug targets for ameliorating autophagy defects [83]. Therefore, drug screens for further potential autophagy modulators could also be carried out for SSADH-D to identify novel disease-specific pathways that could be targeted to enhance autophagy.

## 3. Therapy Options for SSADH Deficiency

### 3.1. Current and Past Clinical Trials in SSADH-D

For three decades, scientists have strived to find a pharmaceutical approach to compensate for the problems caused by a dysfunctional SSADH enzyme, e.g., by influencing the levels of unwanted by-products that stem from the oversupply of GABA. Ideas for curative treatment have also started to emerge, as our understanding of the molecular pathology of the disease is increasing. Some clinical trials targeting the receptors involved in GABA and GHB function have been carried out, with varying success. These treatment concepts and previous clinical trials are summarized in the following paragraphs and in Table 3. Since the treatments and clinical trials have recently been excellently addressed by Vogel and colleagues, here we only shortly summarize the trials and the rationale of these treatments [84].

### 3.2. Clinical Trials Targeting the Neurotransmitter Receptors

The biochemical hallmark of SSADH-D is an increased concentration of GABA, GHB, and other metabolites (see Table 1) in body fluids, such as blood, urine, and cerebrospinal fluid. It is therefore reasonable that the majority of the therapeutic trials have focused on substances that exhibit a direct effect on GABA and GHB receptor subtypes. Preclinical data also exist for a variety of further treatment approaches. These data were mainly generated using the *Aldh5a^−/−^* mouse model that exhibits an early lethal phenotype due to convulsive *status epilepticus* [56,89]. The translation of the results from a mouse model to human disease pathology is limited by the inherent biological differences, such as lissencephalic brains in mice. Therefore, more refined rodent models and in vitro approaches with cellular models closer to humans are required for the transfer of the results from mouse models to humans.

Since most of the therapies with promising results in rodents have shown either no or only low efficacy in clinical trials or exhibit severe adverse effects in humans, there is currently no established guideline for the treatment of SSADH-D. From the group of substances directly interfering with the receptor action, only SGS-742, a GABA_B_ receptor antagonist, is undergoing a double-blind, cross-over, phase 2 clinical trial with human subjects [87]. Preliminary assessment in patients with mild cognitive impairment showed that administration of SGS-742 improved visual information processing, attention, and working memory [85].

The group of classic anti-epileptic drugs, such as vigabatrin and valproic acid, revealed varying outcomes in SSADH-D patients (reviewed in [7]). Vigabatrin, an irreversible inhibitor of GABA transaminase, aims at reducing the high concentrations of GHB by preventing the conversion of GABA to SSA. The drug showed positive effects on behavioral symptoms and refractory epilepsy in only about one third of the patients, and in most cases, the medication has to be discontinued due to a narrowing of the visual field as a side effect of the drug [7]. Moreover, the outcome in patients is inconsistent, as sporadic cases of worsening of symptoms have been reported [100]. Due to the frequent occurrence of visual field impairment associated with the use of vigabatrin, its use should be thoroughly discussed in such vulnerable patient populations.

### 3.3. Further Potential Therapy Options

#### 3.3.1. Enzyme Replacement Therapy: Special Requirements for SSADH-D

Enzyme replacement therapy (ERT) refers to an external substitution of the missing enzyme, usually by delivering a recombinant enzyme by means of injection into the patient’s body. The recombinant enzyme is taken up by the cells and can restore the missing enzyme activity to a varying degree. ERT has been successfully used in various diseases, including several lysosomal storage disorders, such as α-mannosidosis, Pompe disease, and classic late infantile neuronal ceroid lipofuscinosis (cLINCL), just to mention a few examples [101,102,103].

There are some important, enzyme-specific points to consider when developing ERT approaches for a specific disease. The endocytic uptake of the recombinant enzyme is usually dependent on the presence of a suitable receptor protein on the surface of the target cells. In the case of lysosomal enzymes, the delivery is mainly accomplished by the mannose-6-phosphate (M6P) receptors that are ubiquitously expressed. This poses specific requirements for the production of the recombinant enzymes that need to be glycosylated and tagged with M6P to facilitate their uptake into cells. SSADH, on the other hand, is a mitochondrial enzyme whose delivery into its target organelles is coupled with its ribosomal biosynthesis, so that the protein is transferred through the mitochondrial membranes in an unfolded state. Different from lysosomal enzymes, for which uptake mechanism from extracellular fluids exist, SSADH is typically not found outside the cells. Therefore, delivering a recombinant fully folded SSADH enzyme from extracellular space into the mitochondria of patient tissues poses an extra barrier that needs to be crossed in order to obtain a functional improvement and correct cellular delivery.

Another point to consider is the target organ of the therapy. In lysosomal storage disorders, the brain is frequently affected, and the ERT has to reach the CNS in order to be effective. This raises the question about the route of administration, as it may be necessary to deliver the recombinant enzyme directly into the brain/CNS by means of, e.g., intraventricular or intrathecal delivery approaches. This route of delivery is used in the case of Cerliponase alfa (Brineura^®^), the recombinant enzyme used in cLINCL [101]. Peripheral administration usually results in poor brain penetration due to the blood–brain barrier (BBB), but may be sufficient in diseases where mainly peripheral organs are affected. Further potential points to consider are the immunogenicity and stability of the recombinant protein used for the ERT. Since the recombinant enzyme needs to be repeatedly administrated into the patient, and a life-long therapy is required, some patients may develop antibodies against the recombinant protein. This has been observed, e.g., in the case of Gaucher disease [104]. In some cases, the properties of the recombinant enzyme may be improved by chemical modifications, such as PEGylation, as has been shown for phenylalanine ammonia lyase in phenylketonuria [105], or by packing the recombinant enzyme into carriers, such as nanoparticles [106,107].

In SSADH-D, symptoms of the CNS, such as mental impairment and epileptic seizures, are frequently observed, but the exact cause of these symptoms is still under debate. Currently, the main pathogenic role is ascribed to elevated levels of GHB, rather than to GABA. Most of the GHB in our body is produced in the liver, and GHB can efficiently pass the BBB in both directions [108,109]. Thus, in the case of SSADH-D, it may even be sufficient to clear the periphery from GHB by treating mainly the liver, as the excess brain GHB would be expected to equilibrate with the periphery, thus resulting in clearance of the CNS, too.

To date, there is only one report on ERT for SSADH-D in the knockout mouse model [84]. Vogel and coauthors produced a glutathione S-transferase (GST)-tagged SSADH fusion protein in a bacterial expression system. After cleavage of the GST tag, 1 mg/kg of the recombinant SSADH was administrated daily in the SSADH knockout mice intra-peritoneally (i.p.), starting on postnatal day 10. The main outcome of the ERT treatment efficacy was the increased survival of the mice. The authors indeed observed a highly significant increase in survival from a median of 22 days (PBS-treated mice) to 30 days (rSSADH-treated group) in 4 out of 5 mice in this group. In addition, higher mRNA levels of GABA receptor genes were detected in the treated mice. Importantly, the authors demonstrated significantly decreased brain GHB levels in the treated mice, implicating that the peripherally administrated recombinant SSADH enzyme was capable of clearing the brain GHB excess [84]. This is an important indication for a possible ERT-based treatment in SSADH-D patients with recombinant SSADH, especially in terms of the route of administration. However, the recombinant enzyme used in the study evidently does not contain any kind of cell- or brain-delivery sequence that would facilitate its uptake into peripheral cells or the passage through the BBB. Thus, it is unclear if the enzyme would have been capable of entering the cells, let alone the mitochondria. This is an important aspect, since SSADH is not capable of degrading pre-exiting GHB in body fluids and tissues, but it is only able to prevent GHB synthesis by metabolizing SSA into succinate, which is only possible if SSADH is localized in the cells and mitochondria. Therefore, further studies with alternative enzyme delivery approaches should be carried out before this therapy can be translated into clinical applications. However, the data of Vogel et al. build an important cornerstone for ERT in SSADH-D, as they show that a reduction of brain GHB concentration may be obtained by peripheral treatment with recombinant SSADH [84].

#### 3.3.2. Gene Therapy

Already in 2004, Gupta et al. described an adenovirus (AV)-mediated strategy for gene therapy in SSADH-D [110]. Using the SSADH knockout mice, the authors probed two different routes of administration of the virus. Intraperitoneal administration of 10^8^–10^11^ virus particles by postnatal day 10 resulted in a reduction of liver GHB but had no significant impact on brain or serum GHB concentrations. Retro-orbital injection of 10^11^ virus particles at day 13 was shown to reduce the GHB concentration in the periphery (liver, kidney, serum) and in the brain, suggesting that this route may have resulted in CNS penetration. Both viral transduction routes increased the liver SSADH activity up to 20% of the wildtype level, and the survival rates of the treated mice were improved, with i.p. administration resulting in better survival rates [110]. However, the observed effects on GHB concentration and enzyme activity appeared to be quite transient, which might be due to the high immunogenicity of AV proteins and a consequent elimination of the virus by the host immune system. Despite their capacity to carry large recombinant DNA fragments, adenoviral vectors are not state-of-the-art for human gene therapy anymore, mainly due to their high immunogenicity and highly transient transgene expression in dividing cells. However, the study of Gupta and colleagues has been important in demonstrating that gene therapy is capable of reducing GHB and appears to increase the survival in the SSADH-D animal model [110]. Importantly, these data also imply that it may be sufficient to treat peripheral organs, such as the liver, to treat the disease.

Adenoviral vectors have increasingly been replaced by other viral carriers, such as adeno-associated viruses (AAV) and lentiviruses. Recent advances in gene therapy and the development of a number of gene delivery vehicles based on AAV and lentiviruses have boosted preclinical studies of gene therapy in various genetic diseases, and are currently culminating in numerous clinical trials on gene therapy that have started or are about to start. In fact, the Food and Drug Administration (FDA) has recently approved two AAV-based gene therapy vehicles, Luxturna for the treatment of retinitis pigmentosa and Zolgensma for spinal muscular atrophy (reviewed in [111,112]). The next couple of years will hopefully result in the development of gene therapy approaches for various so far untreatable disorders. For the general developments in AAV-mediated gene therapy, we would like to refer the reader to a recent review by Wang and colleagues [113].

In the case of SSADH-D, gene therapy faces similar problems as the ERT approach: mitochondrial enzymes, such as SSADH, are usually not secreted, and cross-correction is thus unlikely to occur at the level that would substantially correct the deficiency in cells not directly targeted by the therapy vehicle. On the other hand, relatively efficient transduction of the liver can be obtained with certain AAV-based vectors [114,115,116,117]. Therefore, if a substantial fraction of the hepatocytes can be corrected by gene therapy, this may result in a large degree of reduction of GHB production, thus lowering the GHB concentration both in the periphery and consequently also in the brain. Since the therapy-induced reduction of GHB in the periphery may be a key issue for the development of future therapies for SSADH-D, it would be important to further study the contribution of elevated GHB to the neurotoxic effects in SSADH-D patients.

#### 3.3.3. Small Molecules: Pharmacological Chaperones and Read-Through Drugs

In SSADH-D, patients exhibit a variety of gene defects, including missense and nonsense variants. Although many of these variants are found in a small number of patients, sometimes only in a single family, some defects, such as the substitutions Cys93Phe or Gly409Asp, have been observed in a number of seemingly unrelated families of different ethnic origin. Similarly, some nonsense variants, such as Trp204X, are present in several patients. Therefore, personalized therapy approaches for specific variants, which are suitable for a number of patients, could be developed for SSADH-D.

Small molecules, such as pharmacological chaperones (PCs) for missense variants and read-through therapies for nonsense variants, have been developed for other diseases and may also provide novel therapeutic means for SSADH-D. Below, we will briefly summarize these strategies and give examples of their use in other diseases. For a more detailed review on substances used for these purposes, we recommend the reviews by Liguori et al. and Nagel-Wolfrum et al. [118,119].

One approach to correct folding defects caused by disease variants and to treat the respective disease is the use of PCs, also known as pharmacoperones [120]. In general, PCs are small molecules that bind to their target proteins and stabilize them. They rescue the function of their targets by facilitating the correct folding, oligomerization, and processing, as well as subsequent trafficking of the proteins to their final destination (e.g., lysosomes). In order to be able to do this, the PCs need to bind in a target-selective fashion. PCs are usually not capable of rescuing every single target molecule synthesized in the cells, so that only a fraction of the target proteins will be correctly folded due to binding to the PC. However, this can already lead to a considerable therapeutic benefit, especially in diseases such as lysosomal storage disorders, where a modest increase in the functional protein is enough to produce a therapeutic effect [120,121].

PCs are mutation specific and are promising only for some missense variants. Therefore, these substances usually cannot be used for all patients with a given disease, but only for those whose variant is expected to respond to the PC. In all cases, the efficacy of a PC should be tested beforehand in suitable cell culture models, e.g., primary patient cells, for each individual variant. In some diseases, one particular variant is the predominant one found in most patients. In the case of aspartylglucosoaminuria (AGU), the most common variant, AGU_Fin-major_, is responsive to PCs, while other missense variants (e.g., R116W, S72P) do not benefit from the same PC substances [122,123].

PC therapy holds great promise for the treatment of numerous protein misfolding diseases, but the identification of a suitable PC for a mutated protein is the most challenging part of this treatment approach (reviewed in [124]). In case of misfolded enzymes, most PCs bind to the catalytic site of the enzyme and might even inhibit the activity of the native enzyme. Therefore, in order to avoid inhibitory effects, such PCs should optimally bind in a reversible manner or they need to have a short half-life. In contrast to enzymes and many viral vectors, some PCs are able to cross the BBB, which makes them especially useful for diseases that show a CNS pathology.

According to the results of large-scale sequencing projects, 11% of all disease-causing gene defects are nonsense variants that result in an in-frame premature termination codon (PTC) [125]. Hence, special therapies targeting this type of variant, i.e., “nonsense suppression therapies”, have the potential to provide treatment for a substantial number of patients suffering from very different diseases. The first substances known to suppress PTCs were the aminoglycoside antibiotics G418 and gentamicin [126,127]. For gentamicin, clinical trials have produced variable results [128,129,130,131], as it does not seem to be efficient for all patients. Long-term administration of aminoglycosides is not feasible due to their strong side effects, such as ototoxicity and kidney damage [132,133]. The varying effects of gentamicin may be explained by the fact that gentamicin is not a pure substance, but is composed of different major and minor gentamicin components. In 2017, the minor component gentamicin B1 was postulated to be responsible for the nonsense-suppression effect of gentamicin, but this paper was later retracted due to problems with the identity of the gentamicin component used in this study [134]. In 2018, the minor gentamicin component X2 was found to be the most active read-through component of gentamicin [135].

In 2007, high-throughput screens for new compounds with nonsense-suppression activity identified PTC124 (Ataluren) as the most promising substance [136]. Ataluren has meanwhile been used in several clinical trials in cystic fibrosis, Duchenne muscular dystrophy, and dystrophinopathy, and it showed some positive effects [137,138,139,140]. The varying efficacy of Ataluren in different diseases may at least in part be explained by the different amounts of residual mRNA that is present in the patient tissues. Depending on the type and location of the PTC, mRNAs harboring nonsense variants may undergo nonsense-mediated decay (NMD), leading to a drastically reduced amount of available mRNA. Hence, NMD inhibition in combination with the PTC suppression will increase the pool of available mRNAs for read-through (reviewed in [141]).

Amlexanox is so far the only substance that combines NMD inhibition and translational read-through [142]. Due to its known anti-inflammatory effects, it has been used for decades in the treatment of other conditions, such as ulcerous lesions of the oral mucosa [143]. In cell culture, amlexanox was able to rescue the expression of functional proteins from mRNAs containing nonsense mutations for p53, dystrophin, aspartylglucosaminidase, and type VII collagen [123,142,144]. Further potentially efficacious read-through agents include the nucleoside analog clitocine and components of fungi extracts that were identified in a recent drug screen [145,146]. Interestingly, the findings of Akaboshi and colleagues suggested that nonsense variants, especially Trp204X and Arg412X substitutions, are frequently present in SSADH-D [38]. Thus, read-through therapies should be tested for nonsense variants resulting in SSADH-D.

## 4. Future of SSADH-D Research and Role of Patient Organizations

### 4.1. Current Tools for Identifying Patients and Patient Registries

As stated above, one major problem in the case of SSADH-D is the difficulty in obtaining the correct diagnosis and identifying as many patients as possible. This will be especially important when novel therapy options hopefully become available in the next years. Fast identification of patients could be obtained by international registries. The International Working Group on Neurotransmitter Related Disorders (iNTD), consisting of 44 partners from 27 different countries, established a web-based registry for inherited neurotransmitter defects, including defects of GABA metabolism [147]. Today, the initiative represents the first longitudinal register for neurotransmitter disorders and enables a systematic gathering of data in very high quality. So far, the registry contains data from more than 350 patients. The iNTD collaborates with other networks, such as MetabERN [148], and is part of the Natural History Study of Patients with SSADH-D (ClinicalTrials.gov Identifier: NCT03758521, [149]). The purpose of this multicentric, multinational approach is to collect a large body of clinical data on SSADH-D in order to gain a deeper understanding of the disease progress, symptoms, and the molecular consequences. Furthermore, novel biomarkers that can be used in monitoring the disease progress and potential therapy success are also searched for. These data will be essential when considering the approval of future therapy approaches by the national authorities.

In addition to identifying patients through registries and patient organizations, improved diagnosis of new SSADH-D cases is highly important. Nowadays, *ALDH5A1* is included in gene panels for epilepsy diagnostics, and many new cases were identified during the last years. However, a large fraction of patients is primarily identified and diagnosed by pediatricians or child neurologists who may not be part of a larger clinical network or a specialized center for rare diseases. Therefore, a highly heterogeneous disease, such as SSADH-D, might go unnoticed for years. The patients are thus not registered and do not have contact with a patient organization to obtain peer support. Therefore, providing the clinicians with information about SSADH-D as a disease, about the diagnostic means and potential therapies, will not only ensure proper care of the patients, but may also result in improved identification of new cases.

Newborn screening is currently not practiced for SSADH-D. However, as soon as novel (targeted) therapies become available, it would be important to include this disease among those that can be diagnosed as early in childhood as possible, in order to make sure that the damage caused by the disease is kept at minimum by starting the therapy as early as possible. Very recently, methods addressing the possibility of newborn detection of SSADH-D from dried blood spots have been developed, paving the way for a screening approach as soon as therapies are available [28].

### 4.2. Roles of Patient Advocacy Organizations in Raising Awareness and Supporting Research

Correct diagnosis is the first key to treating rare diseases. Only when the disease is understood at the molecular level, can the clinicians treat, researchers investigate, and patient advocacy organizations (PAOs) counsel patients regarding further diagnostic investigations and provide support to find appropriate healthcare. Although a particular rare disease may affect only relatively few patients, the number of different rare diseases is currently estimated to be more than 7000, altogether affecting millions of people. The challenges faced by patients include being correctly diagnosed, informed about possible treatments, and receiving proper care and medication. Clinicians, on the other hand, are often confronted with unspecific symptoms, a lack of diagnostic screening instruments, and limited access to relevant resources to assist patients with rare diseases. Researchers, on the other hand, face the problem of obtaining funding and sufficiently large patient cohorts for clinical trials [150]. Furthermore, the availability of validated samples from patients with a specific disease is usually low, limiting the possibilities to use these samples for research work.

General information about a disease is provided through comprehensive databases, such as Orphanet [151]. Further professional support comes from large umbrella organizations, such as EURORDIS (Rare Diseases Europe) [152] in Europe and NORD (National Organization for Rare Disorders) [153] in the USA. Both organizations play an important role in the drug development process, but further key businesses are advocacy for incentives and patient empowerment. Disease-specific PAOs in turn are significantly smaller, often national patient networks and advocacy groups, such as SSADH-Defizit e.V. [154] in Germany, De Neu [155] in Spain, and the SSADH Association [156] in the USA. These disease-specific patient organizations can establish a more direct contact between families, researchers, and clinicians. PAOs also provide information on the disease itself and its consequences in daily life, establishing a platform for exchange with other patients. This is referred to as “peer support” and is the key business of PAOs.

Generally, the funding of research on human diseases stems from government agencies (around 30%) and companies (60%–65%), with only 5% coming from private resources. In rare disease research, however, non-profit organizations are important sources of funding. For example, in the case of Batten disease, nearly 40% of the funding stems from PAOs, while academic research institutions provide 16% and companies less than 5% of the funding [157]. This is currently also true for SSADH-D. Thus, PAOs for SSADH-D have already promoted impressively effective seed projects in laboratory-based research as well as clinical trials.

### 4.3. Future Challenges in SSADH-D

As discussed above, the PAOs have succeeded in attracting further research groups to join the battle against SSADH-D, and a number of novel approaches that directly target SSADH-D at the molecular level are currently under investigation. In SSADH-D, there are no major disease variants that would be present in the majority of patients, but most patients have their own combination of genetic *ALDH5A1* variants. Therefore, personalized therapies may have to be developed for single families or even just for a single patient. This requires a detailed characterization of the molecular consequences of the variants in preclinical laboratory studies, followed by a clinical trial with as little as one patient. Although such personalized and variant-specific approaches based on drug repurposing are promising in rare diseases (see, e.g., [122,123]), therapies aiming at benefiting a larger group of patients should also be developed. In the case of SSADH-D, ERT and gene therapy are valid options, despite the limitations due to the nature of the SSADH enzyme (see Section 3.3). For the approval of such experimental therapies by the authorities and for the development of suitable biomarkers, the Natural History Study in SSADH-D is of vital importance [149]. The authors are confident that within the next five years, we will see new hope emerging for the treatment of SSADH-D since researchers are joining forces to battle this disease.

## Figures and Tables

**Table 1:** Accumulation of potentially harmful GABA metabolites in the body fluids of SSADH-D patients. Modified from [8].

Metabolite	Urine (mmol/mol Creatinine)	Plasma (µM)	CSF (µM)
GHB	34–514 (Ref. < 10) ^1^	26–533 (Ref. < 3)	116–1110 (Ref. < 3)
GABA	n.d. ^2^	n.d.	13.6–22.4 (Ref. < 12)
SSA	3–20 (Ref. < 2)	n.d.	1280–2570 nmol/L (Ref. < 10 nmol/L)
D-2-hydroxyglutaric acid	22–102 (Ref. < 18)	n.d.	04–4.7 (Ref. < 0.3)
Homocarnosine	n.d.	3.1–7.6 (Ref. < 1)	14.8–41 (Ref. < 10)
Guanodinobutyrate	4.6–35 (Ref. < 5.8)	n.d.	0.04–0.32 (Ref. < 0.03)

^1^ Ref: reference value; ^2^ n.d.: not determined.

**Table 2:** Selected missense variants of the human *ALDH5A1* gene that have been associated with SSADH-D. Numbering of the genetic variants is based on the human isoform 2 (NM_001080.3).

Genetic Variant	Amino Acid Change	Domain ^1^	Remark, Severity ^2^	Ref
c.106G>C	Gly36Arg	MTS	Mild	[36,38]
c.278G>T	Cys93Phe	NADB	Several families, severe	[38]
c.526G>A	Gly176Arg	Oligom.	Severe, conserved residue	[39]
c.527G>A	Gly176Glu	Oligom.	Conserved residue	[48]
c. 538C>T	His180Tyr	Oligom.	Mild, exacerbating	[36,38,39]
c.545C>T	Pro182Leu	Oligom.	Mild, exacerbating	[36,38]
c.691G>A	Glu231Lys	NADB	Conserved residue	[48]
c.668G>A	Cys223Tyr	NADB	Severe	[38]
c. 709G>T	Ala237Ser	NADB	Mild, exacerbating?	[36,38,39]
c.698C>T	Thr233Met	NADB	Severe	[38]
c.763A>G	Asn255Asp	NADB	Several families, severe	
c.764A>G	Asn255Ser	NADB	Intermediate	[38]
c.800T>G	Val267Gly	NADB	Conserved residue	[48]
c.803G>A	Gly268Glu	NADB	Cofactor binding, severe	[38,42]
c.901A>G	Lys301Glu	NADB	Cofactor binding	[49]
c.1005C>A	Asn335Lys	Catal.	Dynamic loop, severe	[38,42]
c.1115A>G	Asn372Ser	Catal.	Not characterized	[36]
c.1145C>T	Pro382Leu	Catal.	Severe	[38]
c.1145C>A	Pro382Gln	Catal.	Not characterized	[38]
c.1216G>A	Val406Ile	Catal.	Not characterized	[36]
c.1226G>A	Gly409Asp	Catal.	Severe	[38,47]
c.1267A>T	Thr423Ser	Catal.	Mild, exacerbating?	[39]
c.1498G>C	Val500Leu	Catal.	Substrate binding, severe	[47]
c.1529C>T	Ser510Phe	NADB	Not characterized	[48]
c.1597G>A	Gly533Arg	Oligom.	Severe	[38]

^1^ MTS = mitochondrial targeting sequence; NADB = NAD binding domain; Oligom. = oligomerization domain; Catal. = catalytic domain; ^2^ Severity of the mutation refers to its effect on the SSADH enzyme activity in overexpression systems.

**Table 3:** Summary of the preclinical treatment concepts and clinical trials that have been carried out or are in progress for SSADH-D.

Intervention	Primary Target	Mode of Action	Outcome in Preclinical Models	Clinical Trial and Outcome
SGS-742 [85] CGP-35348 [86]	GABA_B_ receptor	Antagonism	Improvement of epileptiform activity, reduced absence seizures in *Aldh5a^-/-^* mice	Completed, phase 2, 19 patients enrolled [87]
Taurine [88]	Diffuse GABA_A/B_ modulatory receptor effects	Resuscitative effect of an antagonist	Improves survival of *Aldh5a*^−/−^ mice [89]	1 patient, reversal of MRI-documented lesions [90], no effect in TMS [91]
NCS-382 [92,93,94]	GHB receptor	Antagonism	Improves survival of *Aldh5a^−/−^* mice [95], MDCK cells [93]	-
Vigabatrin	GABA transaminase	Inhibition	Improves survival of *Aldh5a^−/−^* mice [56]	Effective in 1/3 of patients. Side effect: narrowing of the visual field [7]
Valproic acid [29,96]	SSADH	Inhibition	-	Increased level of GHB in urine with valproic acid [29]
Rapamycin, Torin, XL-765 [74,78,79,97]	mTORC1/2	mTORC inhibition, induction of autophagy	Improves survival of *Aldh5a^−/−^* mice	-
Tat-Bec1 [97]	Beclin 1	mTORC independent induction of autophagy	Improves survival of *Aldh5a^−/−^* mice, induces modest weight gain	-
Ketogenic diet [98,99]		Neuroprotective effects	Improves survival of *Aldh5a^−/−^* mice [98,99]	

## References

[1] Pearl P.L., Novotny E.J., Acosta M.T., Jakobs C., Gibson K.M. (2003). Succinic semialdehyde dehydrogenase deficiency in children and adults. Ann. Neurol..

[2] Gibson K.M., Jakobs C., Scriver C.R., Beaudet A.L., Sly W.S., Valle D., Childs B., Kinzler K.W., Vogelstein B. (2001). Disorders of beta- and alpha-amino acids in free and peptide-linked forms. The Metabolic and Molecular Bases of Inherited Disease.

[3] Jakobs C., Bojasch M., Monch E., Rating D., Siemes H., Hanefeld F. (1981). Urinary excretion of gamma-hydroxybutyric acid in a patient with neurological abnormalities. The probability of a new inborn error of metabolism. Clin. Chim. Acta.

[4] Gibson K.M., Sweetman L., Nyhan W.L., Jakobs C., Rating D., Siemes H., Hanefeld F. (1983). Succinic semialdehyde dehydrogenase deficiency: An inborn error of gamma-aminobutyric acid metabolism. Clin. Chim. Acta.

[5] Chambliss K.L., Hinson D.D., Trettel F., Malaspina P., Novelletto A., Jakobs C., Gibson K.M. (1998). Two exon-skipping mutations as the molecular basis of succinic semialdehyde dehydrogenase deficiency (4-hydroxybutyric aciduria). Am. J. Hum. Genet..

[6] Benke D., Mohler H. (2018). Impact on GABA systems in monogenetic developmental CNS disorders: Clues to symptomatic treatment. Neuropharmacology.

[7] Vogel K.R., Pearl P.L., Theodore W.H., McCarter R.C., Jakobs C., Gibson K.M. (2013). Thirty years beyond discovery—Clinical trials in succinic semialdehyde dehydrogenase deficiency, a disorder of GABA metabolism. J. Inherit. Metab. Dis..

[8] Malaspina P., Roullet J.B., Pearl P.L., Ainslie G.R., Vogel K.R., Gibson K.M. (2016). Succinic semialdehyde dehydrogenase deficiency (SSADHD): Pathophysiological complexity and multifactorial trait associations in a rare monogenic disorder of GABA metabolism. Neurochem. Int..

[9] Pearl P.L., Gibson K.M., Cortez M.A., Wu Y., Carter Snead O., Knerr I., Forester K., Pettiford J.M., Jakobs C., Theodore W.H. (2009). Succinic semialdehyde dehydrogenase deficiency: Lessons from mice and men. J. Inherit. Metab. Dis..

[10] Pearl P.L., Gibson K.M., Acosta M.T., Vezina L.G., Theodore W.H., Rogawski M.A., Novotny E.J., Gropman A., Conry J.A., Berry G.T. (2003). Clinical spectrum of succinic semialdehyde dehydrogenase deficiency. Neurology.

[11] Pearl P.L., Hartka T.R., Taylor J. (2006). Diagnosis and treatment of neurotransmitter disorders. Curr. Treat. Options Neurol..

[12] Knerr I., Gibson K.M., Murdoch G., Salomons G.S., Jakobs C., Combs S., Pearl P.L. (2010). Neuropathology in succinic semialdehyde dehydrogenase deficiency. Pediatr. Neurol..

[13] Gordon N. (2004). Succinic semialdehyde dehydrogenase deficiency (SSADH) (4-hydroxybutyric aciduria, gamma-hydroxybutyric aciduria). Eur. J. Paediatr. Neurol..

[14] Gibson K.M., Christensen E., Jakobs C., Fowler B., Clarke M.A., Hammersen G., Raab K., Kobori J., Moosa A., Vollmer B. (1997). The clinical phenotype of succinic semialdehyde dehydrogenase deficiency (4-hydroxybutyric aciduria): Case reports of 23 new patients. Pediatrics.

[15] Bak L.K., Schousboe A., Waagepetersen H.S. (2006). The glutamate/GABA-glutamine cycle: Aspects of transport, neurotransmitter homeostasis and ammonia transfer. J. Neurochem..

[16] Kolker S. (2018). Metabolism of amino acid neurotransmitters: The synaptic disorder underlying inherited metabolic diseases. J. Inherit. Metab. Dis..

[17] Bay T., Eghorn L.F., Klein A.B., Wellendorph P. (2014). GHB receptor targets in the CNS: Focus on high-affinity binding sites. Biochem. Pharmacol..

[18] Tillakaratne N.J., Medina-Kauwe L., Gibson K.M. (1995). gamma-Aminobutyric acid (GABA) metabolism in mammalian neural and nonneural tissues. Comp. Biochem. Physiol. A Physiol..

[19] Auteri M., Zizzo M.G., Serio R. (2015). The GABAergic System and the Gastrointestinal Physiopathology. Curr. Pharm. Des..

[20] Wang Y.Y., Sun S.P., Zhu H.S., Jiao X.Q., Zhong K., Guo Y.J., Zha G.M., Han L.Q., Yang G.Y., Li H.P. (2018). GABA regulates the proliferation and apoptosis of MAC-T cells through the LPS-induced TLR4 signaling pathway. Res. Vet. Sci..

[21] Petroff O.A. (2002). GABA and glutamate in the human brain. Neuroscientist.

[22] Siucinska E. (2019). Gamma-Aminobutyric acid in adult brain: An update. Behav. Brain. Res..

[23] Grenier V., Huppe G., Lamarche M., Mireault P. (2012). Enzymatic assay for GHB determination in forensic matrices. J. Anal. Toxicol..

[24] Wernli C., Finochiaro S., Volken C., Andresen-Streichert H., Buettler A., Gygax D., Salomons G.S., Jansen E.E., Ainslie G.R., Vogel K.R. (2017). Targeted screening of succinic semialdehyde dehydrogenase deficiency (SSADHD) employing an enzymatic assay for gamma-hydroxybutyric acid (GHB) in biofluids. Mol. Genet. Metab. Rep..

[25] Toriello H.V. (2012). Approach to the genetic evaluation of the child with autism. Pediatr. Clin. N. Am..

[26] Syndromic Autism Gene Panel. https://www.centogene.com/science/centopedia/syndromic-autism-gene-panel.html.

[27] Attri S.V., Singhi P., Wiwattanadittakul N., Goswami J.N., Sankhyan N., Salomons G.S., Roullett J.B., Hodgeman R., Parviz M., Gibson K.M. (2017). Incidence and Geographic Distribution of Succinic Semialdehyde Dehydrogenase (SSADH) Deficiency. JIMD Rep..

[28] Brown M., Ashcraft P., Arning E., Bottiglieri T., Roullet J.B., Gibson K.M. (2019). Gamma-Hydroxybutyrate content in dried bloodspots facilitates newborn detection of succinic semialdehyde dehydrogenase deficiency. Mol. Genet. Metab..

[29] Shinka T., Ohfu M., Hirose S., Kuhara T. (2003). Effect of valproic acid on the urinary metabolic profile of a patient with succinic semialdehyde dehydrogenase deficiency. J. Chromatogr. B Anal. Technol. Biomed. Life. Sci..

[30] Vanadia E., Gibson K.M., Pearl P.L., Trapolino E., Mangano S., Vanadia F. (2013). Therapeutic efficacy of magnesium valproate in succinic semialdehyde dehydrogenase deficiency. JIMD Rep..

[31] Pearl P.L., Parviz M., Vogel K., Schreiber J., Theodore W.H., Gibson K.M. (2015). Inherited disorders of gamma-aminobutyric acid metabolism and advances in ALDH5A1 mutation identification. Dev. Med. Child. Neurol..

[32] Gropman A. (2003). Vigabatrin and newer interventions in succinic semialdehyde dehydrogenase deficiency. Ann. Neurol..

[33] Gibson K.M., Gupta M., Pearl P.L., Tuchman M., Vezina L.G., Snead O.C., Smit L.M., Jakobs C. (2003). Significant behavioral disturbances in succinic semialdehyde dehydrogenase (SSADH) deficiency (gamma-hydroxybutyric aciduria). Biol. Psychiatry.

[34] Ethofer T., Seeger U., Klose U., Erb M., Kardatzki B., Kraft E., Landwehrmeyer G.B., Grodd W., Storch A. (2004). Proton MR spectroscopy in succinic semialdehyde dehydrogenase deficiency. Neurology.

[35] Chambliss K.L., Zhang Y.A., Rossier E., Vollmer B., Gibson K.M. (1995). Enzymatic and immunologic identification of succinic semialdehyde dehydrogenase in rat and human neural and nonneural tissues. J. Neurochem..

[36] Blasi P., Boyl P.P., Ledda M., Novelletto A., Gibson K.M., Jakobs C., Hogema B., Akaboshi S., Loreni F., Malaspina P. (2002). Structure of human succinic semialdehyde dehydrogenase gene: Identification of promoter region and alternatively processed isoforms. Mol. Genet. Metab..

[37] Kim K.J., Pearl P.L., Jensen K., Snead O.C., Malaspina P., Jakobs C., Gibson K.M. (2011). Succinic semialdehyde dehydrogenase: Biochemical-molecular-clinical disease mechanisms, redox regulation, and functional significance. Antioxid Redox Signal..

[38] Akaboshi S., Hogema B.M., Novelletto A., Malaspina P., Salomons G.S., Maropoulos G.D., Jakobs C., Grompe M., Gibson K.M. (2003). Mutational spectrum of the succinate semialdehyde dehydrogenase (ALDH5A1) gene and functional analysis of 27 novel disease-causing mutations in patients with SSADH deficiency. Hum. Mutat..

[39] Menduti G., Biamino E., Vittorini R., Vesco S., Puccinelli M.P., Porta F., Capo C., Leo S., Ciminelli B.M., Iacovelli F. (2018). Succinic semialdehyde dehydrogenase deficiency: The combination of a novel ALDH5A1 gene mutation and a missense SNP strongly affects SSADH enzyme activity and stability. Mol. Genet. Metab..

[40] Pearl P.L., Wiwattanadittakul N., Roullet J.B., Gibson K.M., Adam M.P., Ardinger H.H., Pagon R.A., Wallace S.E., Bean L.J.H., Stephens K., Amemiya A. (1993). Succinic Semialdehyde Dehydrogenase Deficiency. GeneReviews((R)).

[41] NCBI SNP Search Results for ALDH5A1. https://www.ncbi.nlm.nih.gov/snp/?term=ALDH5A1.

[42] Kim Y.G., Lee S., Kwon O.S., Park S.Y., Lee S.J., Park B.J., Kim K.J. (2009). Redox-switch modulation of human SSADH by dynamic catalytic loop. EMBO J..

[43] Chambliss K.L., Gibson K.M. (1992). Succinic semialdehyde dehydrogenase from mammalian brain: Subunit analysis using polyclonal antiserum. Int. J. Biochem..

[44] Ryzlak M.T., Pietruszko R. (1988). Human brain “high Km” aldehyde dehydrogenase: Purification, characterization, and identification as NAD+ -dependent succinic semialdehyde dehydrogenase. Arch. Biochem. Biophys..

[45] Akiyama T., Osaka H., Shimbo H., Kuhara T., Shibata T., Kobayashi K., Kurosawa K., Yoshinaga H. (2016). SSADH deficiency possibly associated with enzyme activity-reducing SNPs. Brain Dev..

[46] Struys E.A., Verhoeven N.M., Salomons G.S., Berthelot J., Vianay-Saban C., Chabrier S., Thomas J.A., Tsai A.C., Gibson K.M., Jakobs C. (2006). D-2-hydroxyglutaric aciduria in three patients with proven SSADH deficiency: Genetic coincidence or a related biochemical epiphenomenon?. Mol. Genet. Metab..

[47] Leo S., Capo C., Ciminelli B.M., Iacovelli F., Menduti G., Funghini S., Donati M.A., Falconi M., Rossi L., Malaspina P. (2017). SSADH deficiency in an Italian family: A novel ALDH5A1 gene mutation affecting the succinic semialdehyde substrate binding site. Metab. Brain Dis..

[48] Wang P., Cai F., Cao L., Wang Y., Zou Q., Zhao P., Wang C., Zhang Y., Cai C., Shu J. (2019). Clinical diagnosis and mutation analysis of four Chinese families with succinic semialdehyde dehydrogenase deficiency. BMC Med. Genet..

[49] Puttmann L., Stehr H., Garshasbi M., Hu H., Kahrizi K., Lipkowitz B., Jamali P., Tzschach A., Najmabadi H., Ropers H.H. (2013). A novel ALDH5A1 mutation is associated with succinic semialdehyde dehydrogenase deficiency and severe intellectual disability in an Iranian family. Am. J. Med. Genet. A.

[50] Hempel J., Nicholas H., Lindahl R. (1993). Aldehyde dehydrogenases: Widespread structural and functional diversity within a shared framework. Protein Sci..

[51] Karczewski K.J., Francioli L.C., Tiao G., Cummings B.B., Alföldi J., Wang Q., Collins R.L., Laricchia K.M., Ganna A., Birnbaum D.P. (2019). Variation across 141,456 human exomes and genomes reveals the spectrum of loss-of-function intolerance across human protein-coding genes. bioRxiv.

[52] Lancaster M.A., Knoblich J.A. (2014). Generation of cerebral organoids from human pluripotent stem cells. Nat. Protoc..

[53] Jo J., Xiao Y., Sun A.X., Cukuroglu E., Tran H.D., Goke J., Tan Z.Y., Saw T.Y., Tan C.P., Lokman H. (2016). Midbrain-like Organoids from Human Pluripotent Stem Cells Contain Functional Dopaminergic and Neuromelanin-Producing Neurons. Cell Stem Cell.

[54] Kadoshima T., Sakaguchi H., Nakano T., Soen M., Ando S., Eiraku M., Sasai Y. (2013). Self-organization of axial polarity, inside-out layer pattern, and species-specific progenitor dynamics in human ES cell-derived neocortex. Proc. Natl. Acad. Sci. USA.

[55] Monzel A.S., Smits L.M., Hemmer K., Hachi S., Moreno E.L., van Wuellen T., Jarazo J., Walter J., Bruggemann I., Boussaad I. (2017). Derivation of Human Midbrain-Specific Organoids from Neuroepithelial Stem Cells. Stem Cell Reports.

[56] Hogema B.M., Gupta M., Senephansiri H., Burlingame T.G., Taylor M., Jakobs C., Schutgens R.B., Froestl W., Snead O.C., Diaz-Arrastia R. (2001). Pharmacologic rescue of lethal seizures in mice deficient in succinate semialdehyde dehydrogenase. Nat. Genet..

[57] Gibson E.M., Geraghty A.C., Monje M. (2018). Bad wrap: Myelin and myelin plasticity in health and disease. Dev. Neurobiol..

[58] Yalcinkaya C., Gibson K.M., Gunduz E., Kocer N., Ficicioglu C., Kucukercan I. (2000). MRI findings in succinic semialdehyde dehydrogenase deficiency. Neuropediatrics.

[59] Ziyeh S., Berlis A., Korinthenberg R., Spreer J., Schumacher M. (2002). Selective involvement of the globus pallidus and dentate nucleus in succinic semialdehyde dehydrogenase deficiency. Pediatr. Radiol..

[60] Donarum E.A., Stephan D.A., Larkin K., Murphy E.J., Gupta M., Senephansiri H., Switzer R.C., Pearl P.L., Snead O.C., Jakobs C. (2006). Expression profiling reveals multiple myelin alterations in murine succinate semialdehyde dehydrogenase deficiency. J. Inherit. Metab. Dis..

[61] Barcelo-Coblijn G., Murphy E.J., Mills K., Winchester B., Jakobs C., Snead O.C., Gibson K.M. (2007). Lipid abnormalities in succinate semialdehyde dehydrogenase (Aldh5a1-/-) deficient mouse brain provide additional evidence for myelin alterations. Biochim. Biophys. Acta.

[62] Niemi A.K., Brown C., Moore T., Enns G.M., Cowan T.M. (2014). Evidence of redox imbalance in a patient with succinic semialdehyde dehydrogenase deficiency. Mol. Genet. Metab. Rep..

[63] Sgaravatti A.M., Sgarbi M.B., Testa C.G., Durigon K., Pederzolli C.D., Prestes C.C., Wyse A.T., Wannmacher C.M., Wajner M., Dutra-Filho C.S. (2007). Gamma-hydroxybutyric acid induces oxidative stress in cerebral cortex of young rats. Neurochem. Int..

[64] Silva A.R., Ruschel C., Helegda C., Brusque A.M., Wannmacher C.M., Wajner M., Dustra-Filho C.S. (1999). Inhibition of rat brain lipid synthesis in vitro by 4-hydroxybutyric acid. Metab. Brain Dis..

[65] Pearl P.L., Gibson K.M., Quezado Z., Dustin I., Taylor J., Trzcinski S., Schreiber J., Forester K., Reeves-Tyer P., Liew C. (2009). Decreased GABA-A binding on FMZ-PET in succinic semialdehyde dehydrogenase deficiency. Neurology.

[66] Okun J.G., Horster F., Farkas L.M., Feyh P., Hinz A., Sauer S., Hoffmann G.F., Unsicker K., Mayatepek E., Kolker S. (2002). Neurodegeneration in methylmalonic aciduria involves inhibition of complex II and the tricarboxylic acid cycle, and synergistically acting excitotoxicity. J. Biol. Chem..

[67] Chandler R.J., Zerfas P.M., Shanske S., Sloan J., Hoffmann V., DiMauro S., Venditti C.P. (2009). Mitochondrial dysfunction in mut methylmalonic acidemia. FASEB J..

[68] Wajner M., Brites E.C., Dutra J.C., Buchalter M.S., Pons A.H., Pires R.F., Wannmacher L.E., Rosa Junior A., Trindade V.M., Wannmacher C.M. (1988). Diminished concentrations of ganglioside N-acetylneuraminic acid (G-NeuAc) in cerebellum of young rats receiving chronic administration of methylmalonic acid. J. Neurol. Sci..

[69] Schnaar R.L. (2010). Brain gangliosides in axon-myelin stability and axon regeneration. FEBS Lett..

[70] Yu R.K., Nakatani Y., Yanagisawa M. (2009). The role of glycosphingolipid metabolism in the developing brain. J. Lipid Res..

[71] Latini A., Scussiato K., Leipnitz G., Gibson K.M., Wajner M. (2007). Evidence for oxidative stress in tissues derived from succinate semialdehyde dehydrogenase-deficient mice. J. Inherit. Metab. Dis..

[72] Murphy T.C., Amarnath V., Gibson K.M., Picklo M.J. (2003). Oxidation of 4-hydroxy-2-nonenal by succinic semialdehyde dehydrogenase (ALDH5A). J. Neurochem.

[73] Picklo M.J., Montine T.J., Amarnath V., Neely M.D. (2002). Carbonyl toxicology and Alzheimer’s disease. Toxicol. Appl. Pharmacol..

[74] Lakhani R., Vogel K.R., Till A., Liu J., Burnett S.F., Gibson K.M., Subramani S. (2014). Defects in GABA metabolism affect selective autophagy pathways and are alleviated by mTOR inhibition. EMBO Mol. Med..

[75] Menzies F.M., Fleming A., Rubinsztein D.C. (2015). Compromised autophagy and neurodegenerative diseases. Nat. Rev. Neurosci..

[76] Dikic I., Elazar Z. (2018). Mechanism and medical implications of mammalian autophagy. Nat. Rev. Mol. Cell Biol..

[77] Khandia R., Dadar M., Munjal A., Dhama K., Karthik K., Tiwari R., Yatoo M.I., Iqbal H.M.N., Singh K.P., Joshi S.K. (2019). A Comprehensive Review of Autophagy and Its Various Roles in Infectious, Non-Infectious, and Lifestyle Diseases: Current Knowledge and Prospects for Disease Prevention, Novel Drug Design, and Therapy. Cells.

[78] Vogel K.R., Ainslie G.R., Jansen E.E., Salomons G.S., Gibson K.M. (2015). Torin 1 partially corrects vigabatrin-induced mitochondrial increase in mouse. Ann. Clin. Transl. Neurol..

[79] Vogel K.R., Ainslie G.R., Jansen E.E., Salomons G.S., Gibson K.M. (2017). Therapeutic relevance of mTOR inhibition in murine succinate semialdehyde dehydrogenase deficiency (SSADHD), a disorder of GABA metabolism. Biochim. Biophys. Acta Mol. Basis Dis..

[80] Shoji-Kawata S., Sumpter R., Leveno M., Campbell G.R., Zou Z., Kinch L., Wilkins A.D., Sun Q., Pallauf K., MacDuff D. (2013). Identification of a candidate therapeutic autophagy-inducing peptide. Nature.

[81] Rubinsztein D.C., Codogno P., Levine B. (2012). Autophagy modulation as a potential therapeutic target for diverse diseases. Nat. Rev. Drug Discov..

[82] Chauhan S., Ahmed Z., Bradfute S.B., Arko-Mensah J., Mandell M.A., Won Choi S., Kimura T., Blanchet F., Waller A., Mudd M.H. (2015). Pharmaceutical screen identifies novel target processes for activation of autophagy with a broad translational potential. Nat. Commun..

[83] Petcherski A., Chandrachud U., Butz E.S., Klein M.C., Zhao W.N., Reis S.A., Haggarty S.J., Ruonala M.O., Cotman S.L. (2019). An Autophagy Modifier Screen Identifies Small Molecules Capable of Reducing Autophagosome Accumulation in a Model of CLN3-Mediated Neurodegeneration. Cells.

[84] Vogel K.R., Ainslie G.R., Walters D.C., McConnell A., Dhamne S.C., Rotenberg A., Roullet J.B., Gibson K.M. (2018). Succinic semialdehyde dehydrogenase deficiency, a disorder of GABA metabolism: An update on pharmacological and enzyme-replacement therapeutic strategies. J. Inherit. Metab. Dis..

[85] Froestl W., Gallagher M., Jenkins H., Madrid A., Melcher T., Teichman S., Mondadori C.G., Pearlman R. (2004). SGS742: The first GABA(B) receptor antagonist in clinical trials. Biochem. Pharmacol..

[86] Cortez M.A., Wu Y., Gibson K.M., Snead O.C. (2004). Absence seizures in succinic semialdehyde dehydrogenase deficient mice: A model of juvenile absence epilepsy. Pharmacol. Biochem. Behav..

[87] Phase 2 Clinical Trial of SGS-742 Therapy in Succinic Semialdehyde Dehydrogenase Deficiency. https://clinicaltrials.gov/ct2/show/NCT02019667.

[88] Knerr I., Gibson K.M., Jakobs C., Pearl P.L. (2008). Neuropsychiatric morbidity in adolescent and adult succinic semialdehyde dehydrogenase deficiency patients. CNS Spectr..

[89] Gupta M., Hogema B.M., Grompe M., Bottiglieri T.G., Concas A., Biggio G., Sogliano C., Rigamonti A.E., Pearl P.L., Snead O.C. (2003). Murine succinate semialdehyde dehydrogenase deficiency. Ann. Neurol..

[90] Saronwala A., Tournay A.E., Gargus J.J. (2012). Genetic inborn error of metabolism provides unique window into molecular mechanisms underlying taurine therapy. Taurine Health Dis..

[91] Schreiber J.M., Pearl P.L., Dustin I., Wiggs E., Barrios E., Wassermann E.M., Gibson K.M., Theodore W.H. (2016). Biomarkers in a Taurine Trial for Succinic Semialdehyde Dehydrogenase Deficiency. JIMD Rep..

[92] Mehta A.K., Gould G.G., Gupta M., Carter L.P., Gibson K.M., Ticku M.K. (2006). Succinate semialdehyde dehydrogenase deficiency does not down-regulate gamma-hydroxybutyric acid binding sites in the mouse brain. Mol. Genet. Metab..

[93] Vogel K.R., Ainslie G.R., McConnell A., Roullet J.B., Gibson K.M. (2018). Toxicologic/transport properties of NCS-382, a gamma-hydroxybutyrate (GHB) receptor ligand, in neuronal and epithelial cells: Therapeutic implications for SSADH deficiency, a GABA metabolic disorder. Toxicol. In Vitro.

[94] Vogel K.R., Ainslie G.R., Roullet J.B., McConnell A., Gibson K.M. (2017). In vitro toxicological evaluation of NCS-382, a high-affinity antagonist of gamma-hydroxybutyrate (GHB) binding. Toxicol. In Vitro.

[95] Gupta M., Greven R., Jansen E.E., Jakobs C., Hogema B.M., Froestl W., Snead O.C., Bartels H., Grompe M., Gibson K.M. (2002). Therapeutic intervention in mice deficient for succinate semialdehyde dehydrogenase (gamma-hydroxybutyric aciduria). J. Pharmacol. Exp. Ther..

[96] Divry P., Baltassat P., Rolland M.O., Cotte J., Hermier M., Duran M., Wadman S.K. (1983). A new patient with 4-hydroxybutyric aciduria, a possible defect of 4-aminobutyrate metabolism. Clin. Chim. Acta.

[97] Vogel K.R., Ainslie G.R., Gibson K.M. (2016). mTOR inhibitors rescue premature lethality and attenuate dysregulation of GABAergic/glutamatergic transcription in murine succinate semialdehyde dehydrogenase deficiency (SSADHD), a disorder of GABA metabolism. J. Inherit. Metab. Dis..

[98] Nylen K., Velazquez J.L., Likhodii S.S., Cortez M.A., Shen L., Leshchenko Y., Adeli K., Gibson K.M., Burnham W.M., Snead O.C. (2008). A ketogenic diet rescues the murine succinic semialdehyde dehydrogenase deficient phenotype. Exp. Neurol..

[99] Nylen K., Velazquez J.L., Sayed V., Gibson K.M., Burnham W.M., Snead O.C. (2009). The effects of a ketogenic diet on ATP concentrations and the number of hippocampal mitochondria in Aldh5a1(-/-) mice. Biochim. Biophys. Acta.

[100] Matern D., Lehnert W., Gibson K.M., Korinthenberg R. (1996). Seizures in a boy with succinic semialdehyde dehydrogenase deficiency treated with vigabatrin (gamma-vinyl-GABA). J. Inherit. Metab. Dis..

[101] Schulz A., Ajayi T., Specchio N., de Los Reyes E., Gissen P., Ballon D., Dyke J.P., Cahan H., Slasor P., Jacoby D. (2018). Study of Intraventricular Cerliponase Alfa for CLN2 Disease. N. Engl. J. Med..

[102] Borgwardt L., Guffon N., Amraoui Y., Dali C.I., De Meirleir L., Gil-Campos M., Heron B., Geraci S., Ardigo D., Cattaneo F. (2018). Efficacy and safety of Velmanase alfa in the treatment of patients with alpha-mannosidosis: Results from the core and extension phase analysis of a phase III multicentre, double-blind, randomised, placebo-controlled trial. J. Inherit. Metab. Dis..

[103] Bellotti A.S., Andreoli L., Ronchi D., Bresolin N., Comi G.P., Corti S. (2019). Molecular Approaches for the Treatment of Pompe Disease. Mol. Neurobiol..

[104] Pastores G.M., Turkia H.B., Gonzalez D.E., Ida H., Tantawy A.A., Qin Y., Qiu Y., Dinh Q., Zimran A. (2016). Development of anti-velaglucerase alfa antibodies in clinical trial-treated patients with Gaucher disease. Blood Cells Mol. Dis..

[105] Bell S.M., Wendt D.J., Zhang Y., Taylor T.W., Long S., Tsuruda L., Zhao B., Laipis P., Fitzpatrick P.A. (2017). Formulation and PEGylation optimization of the therapeutic PEGylated phenylalanine ammonia lyase for the treatment of phenylketonuria. PLoS ONE.

[106] Del Grosso A., Galliani M., Angella L., Santi M., Tonazzini I., Parlanti G., Signore G., Cecchini M. (2019). Brain-targeted enzyme-loaded nanoparticles: A breach through the blood-brain barrier for enzyme replacement therapy in Krabbe disease. Sci. Adv..

[107] Tosi G., Duskey J.T., Kreuter J. (2019). Nanoparticles as carriers for drug delivery of macromolecules across the blood-brain barrier. Expert. Opin. Drug Deliv..

[108] Bhattacharya I., Boje K.M. (2004). GHB (gamma-hydroxybutyrate) carrier-mediated transport across the blood-brain barrier. J. Pharmacol. Exp. Ther..

[109] Lyon R.C., Johnston S.M., Watson D.G., McGarvie G., Ellis E.M. (2007). Synthesis and catabolism of gamma-hydroxybutyrate in SH-SY5Y human neuroblastoma cells: Role of the aldo-keto reductase AKR7A2. J. Biol. Chem..

[110] Gupta M., Jansen E.E., Senephansiri H., Jakobs C., Snead O.C., Grompe M., Gibson K.M. (2004). Liver-directed adenoviral gene transfer in murine succinate semialdehyde dehydrogenase deficiency. Mol. Ther..

[111] Ziccardi L., Cordeddu V., Gaddini L., Matteucci A., Parravano M., Malchiodi-Albedi F., Varano M. (2019). Gene Therapy in Retinal Dystrophies. Int. J. Mol. Sci..

[112] Keeler A.M., Flotte T.R. (2019). Recombinant Adeno-Associated Virus Gene Therapy in Light of Luxturna (and Zolgensma and Glybera): Where Are We, and How Did We Get Here?. Annu. Rev. Virol..

[113] Wang D., Tai P.W.L., Gao G. (2019). Adeno-associated virus vector as a platform for gene therapy delivery. Nat. Rev. Drug Discov..

[114] Wang L., Bell P., Somanathan S., Wang Q., He Z., Yu H., McMenamin D., Goode T., Calcedo R., Wilson J.M. (2015). Comparative Study of Liver Gene Transfer With AAV Vectors Based on Natural and Engineered AAV Capsids. Mol. Ther..

[115] Wang L., Wang H., Bell P., McCarter R.J., He J., Calcedo R., Vandenberghe L.H., Morizono H., Batshaw M.L., Wilson J.M. (2010). Systematic evaluation of AAV vectors for liver directed gene transfer in murine models. Mol. Ther..

[116] Ginocchio V.M., Ferla R., Auricchio A., Brunetti-Pierri N. (2019). Current Status on Clinical Development of Adeno-Associated Virus-Mediated Liver-Directed Gene Therapy for Inborn Errors of Metabolism. Hum. Gene. Ther..

[117] Baruteau J., Waddington S.N., Alexander I.E., Gissen P. (2017). Delivering efficient liver-directed AAV-mediated gene therapy. Gene. Ther..

[118] Nagel-Wolfrum K., Moller F., Penner I., Baasov T., Wolfrum U. (2016). Targeting Nonsense Mutations in Diseases with Translational Read-Through-Inducing Drugs (TRIDs). BioDrugs.

[119] Liguori L., Monticelli M., Allocca M., Hay Mele B., Lukas J., Cubellis M.V., Andreotti G. (2020). Pharmacological Chaperones: A Therapeutic Approach for Diseases Caused by Destabilizing Missense Mutations. Int. J. Mol. Sci..

[120] Leidenheimer N.J. (2018). Pharmacological Chaperones: Beyond Conformational Disorders. Handb Exp. Pharmacol..

[121] Boyd R.E., Lee G., Rybczynski P., Benjamin E.R., Khanna R., Wustman B.A., Valenzano K.J. (2013). Pharmacological chaperones as therapeutics for lysosomal storage diseases. J. Med. Chem..

[122] Banning A., Gulec C., Rouvinen J., Gray S.J., Tikkanen R. (2016). Identification of Small Molecule Compounds for Pharmacological Chaperone Therapy of Aspartylglucosaminuria. Sci. Rep..

[123] Banning A., Schiff M., Tikkanen R. (2018). Amlexanox provides a potential therapy for nonsense mutations in the lysosomal storage disorder Aspartylglucosaminuria. Biochim. Biophys. Acta Mol. Basis Dis..

[124] Shin M.H., Lim H.S. (2017). Screening methods for identifying pharmacological chaperones. Mol. Biosyst..

[125] Mort M., Ivanov D., Cooper D.N., Chuzhanova N.A. (2008). A meta-analysis of nonsense mutations causing human genetic disease. Hum. Mutat..

[126] Barton-Davis E.R., Cordier L., Shoturma D.I., Leland S.E., Sweeney H.L. (1999). Aminoglycoside antibiotics restore dystrophin function to skeletal muscles of mdx mice. J. Clin. Investig..

[127] Howard M., Frizzell R.A., Bedwell D.M. (1996). Aminoglycoside antibiotics restore CFTR function by overcoming premature stop mutations. Nat. Med..

[128] Clancy J.P., Rowe S.M., Bebok Z., Aitken M.L., Gibson R., Zeitlin P., Berclaz P., Moss R., Knowles M.R., Oster R.A. (2007). No detectable improvements in cystic fibrosis transmembrane conductance regulator by nasal aminoglycosides in patients with cystic fibrosis with stop mutations. Am. J. Respir. Cell Mol. Biol..

[129] Politano L., Nigro G., Nigro V., Piluso G., Papparella S., Paciello O., Comi L.I. (2003). Gentamicin administration in Duchenne patients with premature stop codon. Preliminary results. Acta Myol..

[130] Wilschanski M., Yahav Y., Yaacov Y., Blau H., Bentur L., Rivlin J., Aviram M., Bdolah-Abram T., Bebok Z., Shushi L. (2003). Gentamicin-induced correction of CFTR function in patients with cystic fibrosis and CFTR stop mutations. N. Engl. J. Med..

[131] Woodley D.T., Cogan J., Hou Y., Lyu C., Marinkovich M.P., Keene D., Chen M. (2017). Gentamicin induces functional type VII collagen in recessive dystrophic epidermolysis bullosa patients. J. Clin. Investig..

[132] Jiang M., Karasawa T., Steyger P.S. (2017). Aminoglycoside-Induced Cochleotoxicity: A Review. Front. Cell Neurosci..

[133] Randjelovic P., Veljkovic S., Stojiljkovic N., Sokolovic D., Ilic I. (2017). Gentamicin nephrotoxicity in animals: Current knowledge and future perspectives. EXCLI J..

[134] Baradaran-Heravi A., Niesser J., Balgi A.D., Choi K., Zimmerman C., South A.P., Anderson H.J., Strynadka N.C., Bally M.B., Roberge M. (2018). Retraction for Baradaran-Heravi et al., Gentamicin B1 is a minor gentamicin component with major nonsense mutation suppression activity. Proc. Natl. Acad. Sci. USA.

[135] Friesen W.J., Johnson B., Sierra J., Zhuo J., Vazirani P., Xue X., Tomizawa Y., Baiazitov R., Morrill C., Ren H. (2018). The minor gentamicin complex component, X2, is a potent premature stop codon readthrough molecule with therapeutic potential. PLoS ONE.

[136] Welch E.M., Barton E.R., Zhuo J., Tomizawa Y., Friesen W.J., Trifillis P., Paushkin S., Patel M., Trotta C.R., Hwang S. (2007). PTC124 targets genetic disorders caused by nonsense mutations. Nature.

[137] Bushby K., Finkel R., Wong B., Barohn R., Campbell C., Comi G.P., Connolly A.M., Day J.W., Flanigan K.M., Goemans N. (2014). Ptc124-Gd-007-Dmd Study, G. Ataluren treatment of patients with nonsense mutation dystrophinopathy. Muscle Nerve.

[138] Finkel R.S., Flanigan K.M., Wong B., Bonnemann C., Sampson J., Sweeney H.L., Reha A., Northcutt V.J., Elfring G., Barth J. (2013). Phase 2a study of ataluren-mediated dystrophin production in patients with nonsense mutation Duchenne muscular dystrophy. PLoS ONE.

[139] Kerem E., Konstan M.W., De Boeck K., Accurso F.J., Sermet-Gaudelus I., Wilschanski M., Elborn J.S., Melotti P., Bronsveld I., Fajac I. (2014). Cystic Fibrosis Ataluren Study, G. Ataluren for the treatment of nonsense-mutation cystic fibrosis: A randomised, double-blind, placebo-controlled phase 3 trial. Lancet Respir. Med..

[140] McDonald C.M., Campbell C., Torricelli R.E., Finkel R.S., Flanigan K.M., Goemans N., Heydemann P., Kaminska A., Kirschner J., Muntoni F. (2017). Ataluren in patients with nonsense mutation Duchenne muscular dystrophy (ACT DMD): A multicentre, randomised, double-blind, placebo-controlled, phase 3 trial. Lancet.

[141] Keeling K.M., Wang D., Conard S.E., Bedwell D.M. (2012). Suppression of premature termination codons as a therapeutic approach. Crit. Rev. Biochem. Mol. Biol..

[142] Gonzalez-Hilarion S., Beghyn T., Jia J., Debreuck N., Berte G., Mamchaoui K., Mouly V., Gruenert D.C., Deprez B., Lejeune F. (2012). Rescue of nonsense mutations by amlexanox in human cells. Orphanet J. Rare Dis..

[143] Greer R.O., Lindenmuth J.E., Juarez T., Khandwala A. (1993). A double-blind study of topically applied 5% amlexanox in the treatment of aphthous ulcers. J. Oral Maxillofac. Surg..

[144] Atanasova V.S., Jiang Q., Prisco M., Gruber C., Pinon Hofbauer J., Chen M., Has C., Bruckner-Tuderman L., McGrath J.A., Uitto J. (2017). Amlexanox Enhances Premature Termination Codon Read-Through in COL7A1 and Expression of Full Length Type VII Collagen: Potential Therapy for Recessive Dystrophic Epidermolysis Bullosa. J. Investig. Dermatol..

[145] Benhabiles H., Gonzalez-Hilarion S., Amand S., Bailly C., Prevotat A., Reix P., Hubert D., Adriaenssens E., Rebuffat S., Tulasne D. (2017). Optimized approach for the identification of highly efficient correctors of nonsense mutations in human diseases. PLoS ONE.

[146] Friesen W.J., Trotta C.R., Tomizawa Y., Zhuo J., Johnson B., Sierra J., Roy B., Weetall M., Hedrick J., Sheedy J. (2017). The nucleoside analog clitocine is a potent and efficacious readthrough agent. RNA.

[147] Opladen T., Cortes-Saladelafont E., Mastrangelo M., Horvath G., Pons R., Lopez-Laso E., Fernandez-Ramos J.A., Honzik T., Pearson T., Friedman J. (2016). International Working Group on Neurotransmitter related, d. The International Working Group on Neurotransmitter related Disorders (iNTD): A worldwide research project focused on primary and secondary neurotransmitter disorders. Mol. Genet. Metab. Rep..

[148] MetabERN: European Reference Network for Hereditary Metabolis Disorders. https://metab.ern-net.eu/.

[149] Natural History Study of Patients With Succinic Semialdehyde Dehydrogenase (SSADH) Deficiency. https://clinicaltrials.gov/ct2/show/NCT03758521.

[150] Stoller J.K. (2018). The Challenge of Rare Diseases. Chest.

[151] Orphanet. www.orpha.net.

[152] EURORDIS: Rare Diseases Europe. www.eurordis.org.

[153] NORD: National Organization for Rare Diseases. www.rarediseases.org.

[154] SSADH-Defizit e.V.. www.ssadh.de.

[155] De Neu. www.deneu.org.

[156] SSADH Association. www.ssadh.net.

[157] Stehr F., Forkel M. (2013). Funding resources for rare disease research. Biochim. Biophys. Acta.

